# A cluster randomized controlled trial for measuring the impact of a social norm intervention addressing child marriage in *Pirgacha* in Rangpur district of Bangladesh: study protocol for evaluation of the Tipping Point Initiative

**DOI:** 10.1080/16549716.2022.2057644

**Published:** 2022-04-20

**Authors:** Kausar Parvin, Aloka Talukder, Mahfuz Al Mamun, Sadhvi Kalra, Anne Laterra, Ruchira Tabassum Naved, the Tipping Point Initiative study team

**Affiliations:** a Health Systems and Population Studies Division, icddr,b, Dhaka, Bangladesh; b CARE USA, Atlanta, Georgia, USA

**Keywords:** Child marriage (CM), mixed method, cluster randomized controlled trial, social norms, Bangladesh

## Abstract

Child Marriage (CM) is one of the major developmental concerns in Bangladesh, reporting one of the highest rates of CM (59%) globally. To date, interventions to address CM in Bangladesh have failed to seriously engage with social norms that are important contributors to CM. This paper describes the evaluation design of the Tipping Point Initiative that aims to reduce CM through social norm change and increasing adolescent girls’ agency to voice their rights. The Tipping Point Initiative evaluation trial employs a mixed method design. The quantitative component includes a three-arm Cluster Randomized Controlled Trial design, where Arm 1 receives Tipping Point Program (TPP); Arm 2 receives Tipping Point Program Plus (TPP+), a social norms-enhanced version of TPP; and Arm 3 is the Control. The trial covers 51 clusters (villages) in *Pirgacha*, in Rangpur district, randomized into three study arms (17 per arms). From each cluster, a cohort of 25 adolescent girls aged 12–<16 years were selected randomly for participation in the survey and intervention. Further, a cross-section of adults (six males and six females) were randomly selected from each cluster for survey. Qualitative baseline data were collected from two purposively selected intervention villages in each intervention arm. Thirty In-Depth Interviews, eight Key Informant Interviews and 16 Focus Group Discussion were conducted with adolescent girls, boys, adult women and men. Same strategies have been followed at endline. The intervention was implemented from April 2019 to December 2020. The endline was conducted 10 months after the end of intervention. Intention-to-treat analysis approach will be used for impact assessment. Both narrative analysis and Grounded Theory approach will be employed in analysing qualitative data. The learnings are expected to inform programs and policies regarding what works and does not work to address CM in such social norms intervention in Bangladesh.

## Background and rationale

Child Marriage (CM) bears negative impact on the lives and well beings of girls [1]. Globally, around 650 million females are married before they are 18 [2]. The global rate of CM before age 18 is 40% and before age 15 it is 12% [3]. While the risk of CM in South Asia has declined from 50% to 30% within the last decade, this region still contributes to 44% of the global burden of CM [2]. The adverse impact of CM on girls’ physical and mental health has been well documented [4–8]. CM also inhibits developmental opportunities for girls (e.g. education, income earning, etc.) [8–11]. Child brides are at a higher risk of exposure to intimate partner violence [12–14]. Furthermore, children born to child brides are at 60% greater risk of dying in the first year of life [15]. The economic burden of CM is enormous. Thus, a reduction in child marriage by one standard deviation (16.7 percentage points) could increase annual per capita real GDP growth by 0.66 percentage points in emerging and developing countries [16].

The literature has documented several factors that influence CM such as girls’ education, parent’s education [17], religion [18], rural residence [19], household poverty, dowry, gender inequality, safety, and security [20–22]. CM rates tend to be high in the poorest countries and among the poor who are unable to invest in girls for alternative options [23].

Social norms related to CM have been considered as one of the important determinants of CM [24,25]. Social norms that hinder girls’ education and women’s labour force participation; and norms surrounding girls’ sexuality and purity [26] creates a conducive environment for CM [23]. Human behaviours are guided by social norms. People tend to practice what they believe others do in their community and what others will approve of. At the same time, they tend to avoid deviant behaviour considering the sanctions [27,28]. Thus, despite widespread knowledge regarding adverse consequences of CM, its rate continues to be high in many countries due to prevailing social norms [29]. Therefore, in order to address CM effectively it is imperative to understand and change social norms related to CM in any particular community.

### Child marriage in Bangladesh

Bangladesh has the fourth highest prevalence of CM globally [30], and the highest in South Asia, with 59% of the women aged 20–24 reported being married before the age of 18 [31]. Bangladesh also has one of the highest prevalence of very early marriage, that is, girls married by age 15 (19%). There exist large geographical variations in the rate of CM in this country, ranging between 35% and 70%. Poverty, lower level of education, rural residence, pervasive patriarchal social norms, dowry system, concerns about family reputation are cited as important determinants of CM in Bangladesh [19,32]. Even though Bangladesh has undergone a rapid change in its socio-economic and other developmental and health indicators, there has been little shift in the rate of CM, suggesting that social norms play a stronger role than other risk factors.

In recognition of the need to address high rates of CM, both The Child Marriage Restraint Act, 2017 (Act No. 6 of 2017) and the National Action Plan to End Child Marriage (2018–2030) were developed. Both the government and other civil society organizations and NGOs are investing to eliminate CM. Still, CM is pervasive and the rate of reduction in CM is very slow. Over two decades from 1993 to 2017, the median age at first marriage in this country has risen from 14.1 to only 16.0 [31,33]. Decrease in the prevalence of CM in Bangladesh was the slowest among the South Asian countries [34]. Alarming is the fact that according to Bangladesh Demographic and Health Survey from 2014 to 2017 even these slow declines in CM have most recently stalled [31,35].

### Interventions addressing CM

The Sustainable Development Goals, which have a target to end CM by 2030, have accelerated the efforts of the governments, NGOs and research organizations from different parts of the world in tackling CM through different programs and policies In line with that, a variety of interventions have been implemented and tested in different countries including Bangladesh, India, Nepal, Ethiopia, Kenya and Malawi. Common intervention components have included girls’ education, livelihoods/conditional cash transfer, empowerment, and community mobilization [36–41]. There have been evaluations, but not all of them were evaluated rigorously [42–44], which leads to missed opportunities to produce evidence on what works in reducing CM and what does not.

Within Bangladesh, a few promising interventions have been implemented. A Cluster Randomized Controlled Trial on CM showed that financial incentives to delay marriage were successful to reduce the likelihood of CM by 21%. A combination of financial incentive and empowerment or empowerment alone, however, did not show an effect on CM [45]. Another Cluster Randomized Controlled Trial conducted in southern Bangladesh reports that support in education, promotion of livelihood skills, and gender sensitization interventions, each implemented separately, were effective in reducing CM [40].

Although these interventions proved effective, they did not include any social norm change component, an important root cause of CM and thus, they risk not being sustainable. As pointed out by Kalamar [46], Lee-Rife et al. [47], and Cislaghi [25] lack of understanding of social norms and how to change them effectively impede the development of effective and sustainable CM prevention programs.

The Phase 1 of Tipping Point Initiative (TPI) used a participatory and developmental evaluation approach to identify the social norms that perpetuate CM in Bangladesh and Nepal and worked with communities to drive social change [48,49]. Based on the success of phase 1, TPI Phase 2, an integrated social norms intervention was designed by CARE to address child marriage through a focus on building adolescent girls’ agency, creating supporting relations and transforming norms driving CM. This paper presents the study design of the TPI in Bangladesh.

### Tipping point initiative (TPI) in Bangladesh

TPI is designed to address the root causes of CM. The project aspires to address the communities’ social norms that restrict the lives and roles of girls and uphold the practice of CM. TPI’s approach focuses on synchronized engagement with different participant groups to promote the rights of adolescent girls through community-level programming. TPI developed two holistic implementation packages, Tipping Point Program (TPP) and Tipping Point Program Plus (TPP+), following a multi-year phase of formative research, exploration, and community-action research to ensure that the packages were well tailored to address the root causes of CM in these specific communities. The resulting synchronized approaches are rooted in challenging social expectations and repressive norms and promoting girl-driven movement-building and activism; components designed to help adolescent girls to find and collectively step into spaces to engage with and tackle inequality. The TPI implements and tests two 17-months intervention packages that include the following key components. As part of the ‘core’ program package, the TPP includes the following:

- Weekly sessions with adolescent girls, adolescent boys, and mothers and fathers of adolescents to separately participate in a curriculum that explores social norms related to gender and CM and adolescent sexual health and rights. Girls Group members also participate in sessions around financial literacy and Village Savings and Loan Associations and leadership empowerment, collective action and civic participation

An enhanced package, TPP+, includes the following components in addition to those outlined above:

- Engagement with religious leaders, local government, Union Parishad and community-level influencers through quarterly discussion and dialogues around CM.
- Quarterly intergroup dialogue sessions that bring core participant groups together in facilitated dialogue and reflection

- Identification and training of selected Girl Activist Leaders in campaigning and activism. Once trained, these leaders are linked to other Girls’ Groups and have mentorship to plan and execute their own activities around issues important to them.
- Activist training for selected boys, fathers, and mothers of adolescent girls to build their capacity as allies and supporters of girls’ activism.
- Quarterly community-wide social norms activities organized and led by Girls’ Group members around themes, such as mobility, menstruation, dowry, gendered division of labour, family honour, sexual harassment, and girls’ aspirations.

## Methods

### Aims and objectives of the study

The study aims to evaluate the impacts of the TPP and TPP+ models on CM. The specific objectives are to:

1. Assess the impact of the TPP intervention on reducing child marriage among the adolescent girls participating in TPP.
2. Assess the impact of TPP+ intervention on reducing child marriage among the adolescent girls participating in TPP+.
3. Assess the additional impact of the emphasized social norms change model, TPP+, on child marriage over the impact of the core model, TPP.

### Trial design

**Figure 1.**
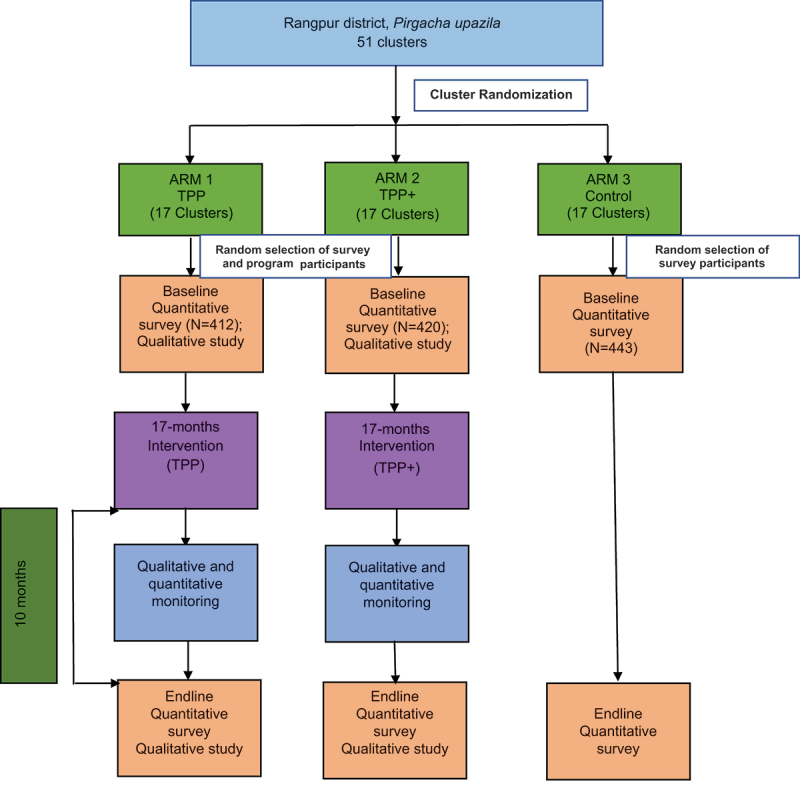
Trial design.

The Tipping Point evaluation trial employs a mixed-method design, including quantitative and qualitative components. It includes a three-arm Cluster Randomized Controlled Trial design (Figure 1). The arms are as follows:

Arm 1: Tipping Point Program (TPP), designed to enhance adolescent girls’ personal assets, intrinsic and instrumental agency;

Arm 2: Tipping Point Program Plus (TPP+), TPP intervention with additional elements designed to enhance social norms change by engaging community leaders and facilitating girl-led community activities; and

Arm 3: Pure control

This design will enable us to measure the effect of TPP and TPP+ interventions, as well as the effect of the enhanced social norms component over TPP. The effects will be determined by comparing the three arms as follows:

Arm 1 – Arm 3 = Effect of TPP intervention

Arm 2 – Arm 3 = Effect of TPP+ intervention

Arm 2 – Arm 1 = Effect of emphasized social norms change

### Study setting

TP intervention and study covered 51 villages, in purposively selected *Pirgacha upazila* (sub-district) from Rangpur district in Bangladesh. Rangpur was chosen as the prevalence of CM was the highest in this division (85%) and the median age at marriage in this district was the lowest (15 years among women aged 20–49) in the country during project inception [50].

#### Cluster selection and randomization

Villages are considered as clusters or the primary sampling units. Initially, a comprehensive list of villages in *Pirgacha upazila* along with the number of households in each village was prepared following the list obtained from the local statistics office. From the list, 51 villages (17 per arm) were selected through a two-stage approach – identification of clusters and randomization to study arms. First, a village was selected at random from the list of all villages within *Pirgacha upazila*. All villages sharing borders with the first selected village were considered ‘buffer’ villages. We then selected the next village in the north-western direction moving anti-clockwise. In addition to the buffer villages, any village consisting of less than 150 households was excluded to ensure a sufficient number of adolescent girls and boys required to form TP intervention groups. Villages in flood-affected and *char* (i.e. river Islands) areas were also excluded considering difficulties in program implementation in such areas. This procedure was followed until the required number of clusters (51) was achieved. Then, each selected village was randomized into one of the three study arms.

#### Household enumeration

Following cluster selection and randomization a household enumeration exercise was conducted in each of the study villages to collect the socio-demographic information (e.g. sex, age, education, religion, marital status, age at marriage, occupation) needed to form the sampling frame for girl and community surveys. A trained team of data collectors conducted this enumeration using a pre-designed form from 9 January – 18 February 2019. The enumeration team received a 3-day participatory training on the form and field procedures. Enumerators were provided with a household enumeration manual. Special care was taken to provide training on how to collect and check information on age and age at marriage. Enumerators cross checked the date of birth with the birth registration card, if available. Any adult person was eligible to provide information for the enumeration. In most of the cases, the respondent happened to be adult female household members (either the household head or wife of household head).

#### Recruitment of study participants and formation of groups

From the household enumeration data, a list of eligible girls and community members was prepared. Girls were eligible for the study if they were: i) aged 12-<16 years; ii) unmarried; and currently living in the study village. Adult males and females aged 25 or more and currently living in the study village were considered eligible to participate in the community survey.

Simple random samples of 29 eligible girls were drawn from each cluster. We oversampled the girls by 16% to achieve a group size of 25, considering the possibility of refusal. In addition, six eligible adult males and six eligible adult females were selected at random from each village for the community survey. In intervention clusters, the lists of selected girls were provided to the implementing organization, which then approached girls from the list of 29 in random order to invite them to participate in the TPI until they had enrolled the 25 target participants per cluster. These girls were then approached to participate in the study, specifically the baseline survey, by the research team. In control villages, and for the adult community samples in each cluster, the research team directly invited the randomly selected participants to enrol in the study during the baseline survey, administered the consent form and interviewed the individuals.

#### Blinding

No one was blinded to their study arm allocation.

## Trial outcomes

### Primary outcome: child marriage among girls

Child marriage is defined as marriage of a girl before the age of 18 years. CM will be measured among the study participants and the measure will include % of marriage before the age of 18 and 15 years. Comparisons will be made between the intervention and control clusters.

### Secondary outcomes

Secondary outcomes measured are: (1) Intrinsic agency – defined as one’s consciousness of own aspirations, capabilities, and rights [51–53]; (2) Instrumental agency – defined as the action to achieve one’s aspirations and goals [51–53]; (3) Collective agency – defined as the collective action to achieve common goals [51–53]; (4) social norms – defined as the normative expectations and empirical expectations regarding child marriage, and girls’ ability to stand up for their rights [54,55].

The specific secondary outcomes of TP include: (1) Adolescent girl’s self-efficacy in achieving life goals and collective efficacy in preventing CM; (2) Adolescent girl’s aspiration about marriage and education; (3) Adolescent girl’s knowledge about Sexual and Reproductive Health and Rights; (4) Attitudes towards gender and rights among adolescent girls and adult community members; (5) Cohesion among adolescent girl; (6) Communication and negotiation skill of adolescent girls; (7) Adolescent girl’s mobility; (8) Participation in financial activities among adolescent girls; (9) Power relations between adolescent and parents; and (10) Perception of Social norms among adolescent girls and adult community members. The details of the study outcomes are presented in Table 1.

**Table 1.** Primary and secondary outcomes of the Tipping Point impact evaluation in Bangladesh

SL	Outcomes	Indicator/ domain	Target population	Measurement	Questions/Scale used	Number of items	Cronbach Alpha	KMO	Expected direction of change
**Primary outcomes**									
1.	Child marriage	Rate of CM	Girls	Proportion married before 18 years Proportion married before 15 years	-	-	-	-	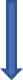
**Secondary outcomes**									
1.	Efficacy	Intrinsic agency – Self-efficacy	Girls	Proportion of girls reported higher self-efficacy (using tertiles of score)	Girls’ perceived confidence in achieving life goals in education, healthcare, mobility, marriage, and income earning	8	0.79	0.80	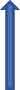
		Collective agency – Collective efficacy	Girls	Proportion of girls reported higher collective efficacy (using tertiles of score)	Questions were framed around collective action involving the community around preventing child marriage, preventing violence against girls, etc.	5	0.83	0.79	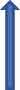
2.	Aspirations regarding marriage and education	Intrinsic agency	Girls	Frequencies of aspirations around education and preferred age at marriage	Questions were framed around their desired level of education, age at marriage	-	-	-	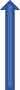
3.	Knowledge regarding Sexual and Reproductive Health and Rights (SRHR)	Intrinsic agency	Girls	Proportion of girls reported correct knowledge on SRHR	Questions were framed around their knowledge about SRHR	-	-	-	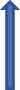
4.	Attitudes regarding gender and rights	Intrinsic agency	Girls	Proportion of girls reported higher gender equitable attitude (using tertiles of score)	Modified version of the Gender-Equitable Men (GEM) Scale [59]. Three sub-scales were constructed for girls	7 items for gender roles	0.70	0.81	
						4 items for attitudes regarding controlling by family members	0.76	0.73	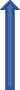
						8 items for attitudes regarding justification of girls beating	0.78	0.82	
			Adult male and female community members	Proportion of adult community members reported higher gender equitable attitude	Modified version of the Gender-Equitable Men (GEM) Scale [59]. Four sub- scales were constructed for adult community members	4 items for gender roles	0.61	0.70	
						4 items for attitudes regarding controlling by family members	0.94	0.80	
						9 items for attitudes regarding justification of girls beating	0.89	0.91	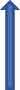
						4 items for gender discrimination	0.67	0.71	
5.	Cohesion	Collective agency	Girls	Proportion of girls demonstrated higher cohesion	The neighborhood cohesion scale [60]	13	0.93	0.95	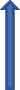
6.	Communication and negotiation	Instrumental agency -Communication skills	Girls	Proportion of girls demonstrated good communication skills	Communication and negotiation scale developed by Liu et al [61]	10	0.72	0.67	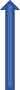
		Intrinsic agency – Confidence regarding negotiation	Girls	Proportion of girls demonstrated higher confidence regarding negotiation	Three questions were asked to measure confidence in negotiating education, marriage and mobility	3	0.72	0.68	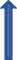
7.	Mobility	Instrumental agency	Girls	Proportion of girls reported higher mobility	Questions were framed around girl’s ability to move certain places	6	0.53	0.69	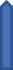
8.	Participation in financial activities	Instrumental agency	Girls	Frequencies of participation in financial activities	Six questions related to their involvement in financial activities were formulated				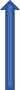
9.	Power relation	Connectedness with parents	Girls	Proportion of girls reported high connectedness	Connectedness was measured by asking several questions about their relations with parents	7	0.77	0.82	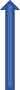
		Gender discrimination within family	Girls	Proportion of girls reported low gender discrimination	Questions were asked about their experience of discrimination within family	5	0.58	0.68	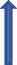
10.	Social norm	Social norms	Girls	Proportion of girls demonstrated positive social norm	around normative expectations regarding girls’ practices	4	0.61	0.69	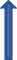
			Adult male and female community members	Proportion of adult community members demonstrated positive social norm	Measured using statements about normative expectations (injunctive norms) regarding girls’ practices and parents’ practices	8	0.71	0.80	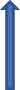

*CM – Child marriage

**KMO – Kaiser-Meyer-Olkin test**

### Quantitative surveys

The quantitative component includes baseline and endline (10 months after the end of intervention) data collection from the same cohort of adolescent girls and separate cross-sectional samples of adult male and female community members in all 51 study villages.

#### Sample size

The sample size for the quantitative component was calculated based on the primary outcome ‘child marriage’ among girls aged between 12 years and below 16. The rate of CM for this age group was unknown. Thus, we assumed a 50% prevalence rate and considered a cluster size of 22 adolescent girls. A life skills intervention in Maharashtra, achieved 19% reduction in CM in the community [38]. Based on this, we assumed a 15% effect size and an Intra-Cluster Correlation of 0.05. Considering 5% significance level and 80% power we required 17 clusters per arm making the total number of clusters 51. Considering a 15% non-response/dropout rate the group size increased to 25 and total sample size increased to 1,275 girls. To assess social norms change we considered child marriage related social norms among the adult community members aged 25 or more as the primary outcome. Since the prevalence of CM related norms is unknown in the study villages, we assumed it to be 50%. Considering a 15% effect size, 5% significance level, 80% power and 5% non-response rate, we required 540 community members from 51 clusters. To ensure participation of both males and females we required six adult males and six adult females from each cluster.

### The baseline quantitative survey

#### Data collection

Two separate questionnaires were developed in English and then translated into Bangla for the girl’s and community surveys. The Bangla version of the questionnaires were pre-tested and cognitive interviews were conducted during 10–14 December 2018 in two villages from Rangpur district, which were outside the TPI study area. Scales not used previously in Bangladesh and questions deemed difficult to comprehend were identified for cognitive interview. Both the Bangla and English versions of the questionnaires were then modified based on the feedback received from the pre-test. The questionnaires were finalized after piloting conducted at the end of the survey team training.

A team of 14 females and two males with graduate degree collected the baseline data. The team was accompanied by four supervisors, two quality control officers and one survey coordinator. The survey team received a 12-day participatory training from 8–29 January 2019 on gender, child marriage, empowerment of women and adolescent girls, survey methods, the questionnaire, research ethics, use of tablets, data collection techniques, field management, and data quality.

The baseline survey was conducted from 2 February – 9 April 2019 using separate pre-designed questionnaires for adolescent girls and community members. Data were collected face-to-face in Bangla by gender-matched interviewers upon receipt of oral assent of adolescent girls and oral consent of their parents in the girls’ survey and oral consent of the participants in the community survey. All the interviews took place in private, in a location convenient for the participants, and used tablets. The survey coordinator was responsible for coordinating survey implementation in the field. The supervisors assigned daily tasks to the interviewers and received feedback at the end of each working day. A selected participant was considered unavailable if the survey team could not reach her/him after three visits during the whole period of data collection.

#### Data quality monitoring

A comprehensive data quality monitoring system was in place. At the first level, the supervisors observed and spot-checked the interviews and provided feedback to the interviewers, if there was any. The researchers made frequent field visits, randomly checked filled-out questionnaires, observed interviews where possible and provided feedback to the survey team. Secondly, the quality control officer checked each completed questionnaire for any inconsistencies and solved the issue in consultation with the interviewers. Further, a computer-based data checking routine was developed and inconsistencies of collected data were checked routinely. Problems identified in the data were communicated to the supervisors through the survey coordinator. The supervisor resolved the problems through discussion with the interviewer if possible. If necessary, the interviewer revisited the respondent and solved the issues by consulting the respondent. Moreover, 5% of the interviews were revisited by the supervisor and they administered a short questionnaire focused mainly on identifying any problems in adhering to ethical guidelines and administering questions on particular topics.

#### Scale construction and reliability checks

In preparation for endline data collection and analysis, the study team validated the scales using the baseline data and the number of items in the scales was reduced using factor analysis. The rotated factor loading of an item against all the suggested factors was checked for non-loading and cross-loading. The cut-off for item loading was set at 0.35. An item was considered as cross-loaded if it loaded under two or more factors. The analysis was rerun excluding the non-loaded and cross-loaded items one by one. This process continued until no non-loaded or cross-loaded item was left. Any factor with less than three items was dropped. Internal consistency (or reliability) of a scale was measured using Cronbach’s alpha. A scale with alpha equal to 0.60 or more was considered acceptable. Scale validity was measured using the Kaiser–Meyer–Olkin (KMO) test. KMO equal to 0.60 or more was considered acceptable.

#### Baseline survey data analysis

The household enumeration data were used to estimate the baseline proportion of child marriage among males (married before age 21) and females (married before age 15, and 18). Using the baseline survey data, the basic background characteristics (e.g. age, education, religion, and ethnic group) of the girl and community samples were compared by arm (control, TPP, and TPP+) to check the arm balance. The arms were considered balanced if there were no or few significant differences in background characteristics.

Descriptive analyses were carried out using the baseline data to report mean score, proportion, tertiles of score for selected outcomes. All group differences were assessed using χ^2^-tests of independence for categorical variables and t-tests for continuous variables. Significance level was set at p < .05 for all bi-variate analyses. The baseline analyses were conducted in STATA 15.

### The endline quantitative study

The same cohort of adolescent girls and separate cross-sectional samples of adult male and female community members were interviewed during the endline, ten months after the end of intervention. The questionnaires were updated to include specific questions on exposure and participation on TPI, exposure and impact of COVID on outcomes. Additionally, items are dropped from scales based on scale validation performed on baseline data. An approach similar to baseline is being followed in interviewing the study participants.

#### Impact evaluation analysis

Intention-to-treat analysis will be used for assessing the impact of the TP program. Thus, all the TP girls enrolled in TPI will be included in the analysis irrespective of their actual level of participation in the intervention activities. The impact of the TP program on primary outcome, child marriage will be assed using hazard ratio while the TP secondary outcomes will be assessed using risk ratios derived from binary regression analyses for measuring change in outcomes in the intervention arms relative to change in the control arm. Mixed-effects models will be constructed with clusters as random effects and time point, intervention group, and time point x intervention interaction as fixed effects. Adjusting for the dependent variables at baseline survey we will obtain a measure of the relative change in intervention group that took place between baseline and endline compared to the change in the control group. The background characteristics for which the differences between study arms are evident, the pre-existing differences will be controlled in subsequent analyses. All analyses will be adjusted for the potential factors associated with the outcomes of interest.

### The qualitative component

The qualitative component includes both baseline and endline data collection from adolescent girls and boys, adult male and female community members and key informants from purposively selected villages in Arm 1 and Arm 2. This will provide an in-depth understanding of the context and nature and pathways of change. Following enumeration, the qualitative sample was drawn on purpose through informal discussions with community members from two villages from each intervention arm, TPP and TPP+. Unmarried adolescent girls and boys aged 12-<16 and currently living in the study villages were selected for In-depth Interviews (IDI) and Focus Group Discussions (FGD); fathers and mothers of adolescents were chosen for FGDs and village residents who were knowledgeable as well as willing to talk about CM in the context of their village were selected for conducting Key Informant Interviews (KII) from each village. The use of three different qualitative techniques of data collection will allow data triangulation. While KIIs and FGDs will provide data on social norms and general practices, IDIs will give us access to in-depth data on individual perceptions, practices and experiences of adolescent girls and boys.

### The baseline qualitative study

Once villages were assigned to the study arms, a total of four villages (two villages from each intervention arm) were purposively selected for baseline qualitative data collection. Four KIIs; 10 IDIs with adolescent girls and five IDIs with adolescent boys; two FGDs with adolescent girls and two with boys; two FGDs with adult women and two with adult men in the community were completed in each arm. The sample distribution by participant categories and by arms have been presented in Table 2.

**Table 2.** Number of focus group discussions (FGD), Key informant interviews (KII), and In-depth interviews (IDI) by participant category and by arm in Rangpur

Sl	Tools and participant category	Arm 1	Arm 2	Total
	KIIs with men	2	2	4
	KIIs with women	2	2	4
	IDIs with adolescent girls (Group members)	10	10	20
	IDIs with adolescent boys (Group members)	5	5	10
	FGDs with adolescent girls (Group members)	2	2	4
	FGDs with adolescent boys (Group members)	2	2	4
	FGDs with adult women/mother (Group members)	1	1	2
	FGDs with adult women/mother (Non-group members)	1	1	2
	FGDs with adult men/father (Group members)	1	1	2
	FGDs with adult men/father (Non-group members)	1	1	2
**Total**		**27**	**27**	**54**

Qualitative data were collected during 26 February – 31 March 2019. A team of five researchers with Masters degrees in Anthropology collected qualitative data. They received a 12-day participatory training during 8–29 January 2019 on gender, research ethics, qualitative research methods, current study and qualitative guides.

Interviews and discussions with participants were conducted in Bengali by gender-matched interviewers. Data were audio recorded upon receipt of verbal approval from the participants and guardians (in case of adolescent participants). All participants agreed to follow-up visits. In some cases, follow-ups were conducted, either face to face or over the phone to fill gaps in the data. Recruitment processes of the participants and general observations of each interview were documented by all the interviewers. The researchers used to take part in a compulsory daily debriefing session at the end of each working day for discussing interesting findings, reviewing field notes and experiences. This process helped the team to scrutinize interesting findings which facilitated an iterative process of data collection. It also helped the researchers to resolve issues or challenges related to the data collection.

#### Baseline qualitative data processing and analyses

The recorded baseline data were transcribed verbatim in Bengali and translated into English. Transcripts were anonymized by removing any information that could identify the participant such as: names of people, their address. The identifying information was kept in a separate file. The accuracy and completeness of the transcripts were examined by listening to a random sample of 20% of the audio-files of the IDIs, KIIs, and FGDs. The study team marked unclear transcripts for transcriber correction and reviewed corrections upon submission. Researchers always went back to the audio files to maintain the accuracy and completeness of the transcripts and translation for all the interviews.

Bengali transcripts were entered into MAXQDA 18 (VERBI 2018) for qualitative data analysis in order to facilitate coding and data analysis. Two members of the research team coded the transcripts using a priori and inductive coding. Inter-coder agreement was achieved during training, where data were first coded jointly; each coder then coded individual data segments, comparing two sets of coding and discussing differences in order to reach an agreement. This procedure was carried out several times until complete coding agreement was achieved. Once coding was completed the data were retrieved by codes for further analyses by themes. CARE’s Social Norms Analysis Plot framework guides thematic analysis, which defines five key elements of a norm: empirical and normative expectations, sanctions, sensitivity to sanctions, and exceptions [56]. The data was subjected to narrative analysis also to gain a better understanding of the norms associated with CM. Repeated discussion took place among the researchers allowing enough scope for examining the data critically, enhancing the rigour of analysis, and reflecting upon the findings.

### The endline qualitative study

The endline data were collected from the same villages using the same sample distribution as baseline. The interview guides were modified to capture exposure and impact of TPI and COVID for data collection and the same analyses technique will be employed.

## Ethical consideration

This study follows the WHO ethical recommendations for researching violence against women [57] and the CIOMS International Guidelines for Ethical Review of Epidemiological Studies [58] for both quantitative and qualitative components. The study (PR#18056) received ethical approval from icddr,b’s Institutional Review Board. Participation in the study was voluntary and all the study participants were included in the study upon their consent. Data were collected in Bangla using face-to-face interviews upon receipt of oral assent and consent of adolescent girls and adult community members, respectively. Oral consent of the parents of adolescent girls’ survey were also sought. Because of the low levels of literacy and concerns regarding confidentiality, verbal consent was obtained from the participants prior to conducting the interviews. All the baseline interviews were conducted in private and in a location convenient for the participants. The participants were forewarned that the data collected will be held in strict confidence and includes questions on highly personal and sensitive topics. The participants were free to terminate the interview at any point, and to skip any questions that s/he does not wish to respond to.

### Participants’ timeline

Selection of clusters (villages) was completed during December 2018 – February 2019. Household enumeration was conducted during January to March, 2019. The study participants were recruited and the baseline data were collected during January to April 2019. As soon as the participants from a village were recruited baseline started in that village while recruitment was continuing in other villages. The implementation of the intervention in the first cluster started in April 2019 once the survey was completed in that cluster, and subsequently in other villages. The implementation of intervention has been completed in December 2020. Though, the intervention was originally designed to be implemented over 18 months, due to COVID-19 pandemic and its associated lock down and related restrictions few sessions were merged together and the intervention was completed over 17 months period. The endline was conducted from November 2021 to January 2022, 10 months after the end of intervention.

## Discussion

The TPI study aims to address CM through addressing social norms. To our knowledge, this study is the first of its kind. As suggested by previous literature [45–47] addressing the root cause of CM is important for tackling CM. The TPI provides an opportunity to evaluate whether and how social norms favouring CM can be addressed for reaching a sustainable solution to the harmful practice of CM. Moreover, it allows us to compare two different approaches to social norm change – one of which focuses on the girls’ empowerment only, while the other combines it with capacity building in leadership and collective action and mobilization and involvement of other stakeholders in the movement against CM.

TPI used a Cluster Randomized Controlled Trial design, which is considered the gold standard for program evaluation. The design will allow us to build evidence regarding the effectiveness of TPI. However, TPI has a few limitations and faced several challenges during the inception, data collection and implementation phases. We discuss these along with the mitigation strategies that were adopted in the project.

### Challenges and limitations

First of all, collecting accurate information on age is always a challenge in countries like Bangladesh where most people do not usually keep track of age, and birth registration is a recent phenomenon. To deal with this issue, we provided intensive training to the household enumerators and data collectors on several strategies to determine age as accurately as possible. For example, recalling any historical events related to the birth like flood, draught, political event, etc. In case of girls, age at menarche was used as an additional check. In these circumstances, it is likely that the participants in household enumeration were not always able to provide correct information on age. Thus, some of the randomly selected eligible respondents from enumeration data were found ineligible when they were screened for their age at the time of the survey. We have replaced these cases with eligible girls. The survey team cross-checked the reported age with the birth registration card, but sometimes such cards were not available.

Secondly, reporting of age at marriage may be biased also in an attempt to avoid disclosing illegal CM. We tried to minimize this bias by calculating the birth year, marriage year and duration of marriage. Thirdly, in intervention clusters the girls who agreed to participate in the program were recruited and interviewed. This may result in possible selection bias. To avoid this bias, in control communities we added a hypothetical question on their willingness to participate in such program. Only three girls mentioned that they would not participate in such program. Thus, it can be said that the girls are not different from those in the intervention communities and minimizes the possibilities of selection bias.

Fourth, reaching the girls was extremely difficult as many were busy for the majority of each day with school, tutoring, and coaching. Therefore, the interviews had to be taken either in the early morning or in the evening. Some adolescent girls had to be interviewed in school with the permission of the authority as it was not possible to access them otherwise. The baseline survey overlapped with the harvest season and thus, some community members had to be interviewed in the field that is not ideal locations for conducting interviews and maintaining privacy was a major concern. However, maximum effort was provided to maintain privacy and confidentiality. No interview was conducted in front of others.

Fifth, the family/community members were curious about the interviews and often wished to observe the process. In this situation, if questions on a sensitive issue was being asked (e.g. on menstruation, aspirations regarding marriage) the interview either switched to a non-sensitive section or stopped the interview, resumed it only when others left. Sixth, adolescent girls felt shy to answer some of the questions, particularly, about reproductive and sexual health. In order to address this, the interviewers were trained to be non-judgemental and friendly. Lastly, the qualitative study was conducted in four villages only. Therefore, the results are not generalizable to all study villages. Moreover, the hard to reach areas were excluded from the study so, findings might not be generalizable to those contexts.

## Conclusion

Addressing CM is crucial for the development of girl’s individual life and as a whole for the nation. If TPI proves effective, it will bear important policy and program implication. Learning from this study will make important contribution in expanding the body of knowledge about what works and what does not work in addressing CM effectively.

## Data Availability

Not Applicable.

## Paper context

We describe the mixed method, randomized controlled trial design to assess the impact of Tipping Point Initiative in addressing child marriage through social norm change interventions. Findings form this study has high potential to inform policy and program on effective interventions to address child marriage in similar context.
